# Excess Death Estimates in Patients with End-Stage Renal Disease — United States, February–August 2020

**DOI:** 10.15585/mmwr.mm7022e2

**Published:** 2021-06-04

**Authors:** Robert Ziemba, Kyle N. Campbell, Tsu-Hsuan Yang, Sara Eve Schaeffer, Kelly M. Mayo, Paul McGann, Shalon Quinn, Jesse Roach, Edwin D. Huff

**Affiliations:** ^1^Health Services Advisory Group, Inc., Tampa, Florida; ^2^iQuality Improvement and Innovation Group, Center for Clinical Standards and Quality, Centers for Medicare & Medicaid Services, Baltimore, Maryland; ^3^Division of Kidney Health, Center for Clinical Standards and Quality, Centers for Medicare & Medicaid Services, Baltimore, Maryland; ^4^Quality Measurement and Value-Based Incentives Group, Center for Clinical Standards and Quality, Centers for Medicare & Medicaid Services, Baltimore, Maryland.

End-stage renal disease (ESRD) is a condition in which kidney function has permanently declined such that renal replacement therapy* is required to sustain life (*1*). The mortality rate for patients with ESRD in the United States has been declining since 2001 (*2*). However, during the COVID-19 pandemic, ESRD patients are at high risk for COVID-19–associated morbidity and mortality, which is due, in part, to weakened immune systems and presence of multiple comorbidities (*3*–*5*). The ESRD National Coordinating Center (ESRD NCC) supports the Centers for Medicare & Medicaid Services (CMS) and the ESRD Networks^†^^,^^§^ through analysis of data, dissemination of best practices, and creation of educational materials. ESRD NCC analyzed deaths reported to the Consolidated Renal Operations in a Web-Enabled Network (CROWNWeb), a system that facilitates the collection of data and maintenance of information about ESRD patients on chronic dialysis or receiving a kidney transplant who are treated in Medicare-certified dialysis facilities and kidney transplant centers in the United States. Excess death estimates were obtained by comparing observed and predicted monthly numbers of deaths during February 1–August 31, 2020; predicted deaths were modeled based on data from January 1, 2016, through December 31, 2019. The analysis estimated 8.7–12.9 excess deaths per 1,000 ESRD patients, or a total of 6,953–10,316 excess deaths in a population of 798,611 ESRD patients during February 1–August 31, 2020. These findings suggest that deaths among ESRD patients during the early phase of the pandemic exceeded those that would have been expected based on previous years’ data. Geographic and temporal patterns of excess mortality, including those among persons with ESRD, should be considered during planning and implementation of interventions, such as COVID-19 vaccination, infection control guidance, and patient education. These findings underscore the importance of data-driven technical assistance and further analyses of the causes and patterns of excess deaths in ESRD patients. 

CROWNWeb^¶^ is the national ESRD patient registry and contains administrative and clinical data submitted by dialysis facilities in the United States (*6*). Dialysis facility admission and discharge records in CROWNWeb for transplant and dialysis patients were accessed to identify decedents. Estimates of excess deaths during the early months of the COVID-19 pandemic (February–August 2020) were expressed as a range based on methodology established by CDC (*7*). The upper limit of excess deaths was defined as the difference between the observed and the predicted number of deaths; the lower limit was defined as the difference between the observed number of deaths and the upper end of a one-sided 95% prediction interval from the model. The predicted number of deaths was calculated using a Poisson model with five variables: year, month, age group, age-group-by-year interaction term, and ESRD Network service area. The month and year variables were added to model the seasonal and secular trends in mortality, which were observed in the data. The model was fit with observations from 2016 to 2019; predictions for 2020 assume that seasonal and secular trends in death rates observed during 2016–2019 were replicated in 2020.** All analyses were conducted using SAS (version 9.4; SAS Institute). This activity was reviewed by CMS and was conducted consistent with applicable federal law and CMS policy.^††^

Excess death estimates during February 1–August 31, 2020, at the ESRD Network service area and national levels were compared with the total ESRD patient population size from February 2020 to allow comparisons between populations. Excess mortality for all ESRD patients was compared using analyses that included dialysis or kidney transplant patients, defined by the last treatment type for each patient before death.^§§^ For each subgroup analysis, a new prediction model was estimated, and the numbers of patients who had a most recent dialysis treatment or kidney transplant before death or before February 1, 2020, were used to calculate the total dialysis or kidney transplant patient population sizes, respectively.

A total of 410,297 decedents were identified in the CROWNWeb data set during January 1, 2016–August 31, 2020, including 60,317 (14.7%) deaths that occurred during February 1–August 31, 2020. Based on the 798,611 patients who were on dialysis or had a kidney transplant as of February 2020, an estimated 8.7–12.9 excess deaths per 1,000 patients occurred among this ESRD population during the early phase of the COVID-19 pandemic, representing 6,953–10,316 excess deaths (Figure 1). Excess deaths at the national level peaked in the early months of the pandemic with a smaller peak in late summer.

**FIGURE 1:**
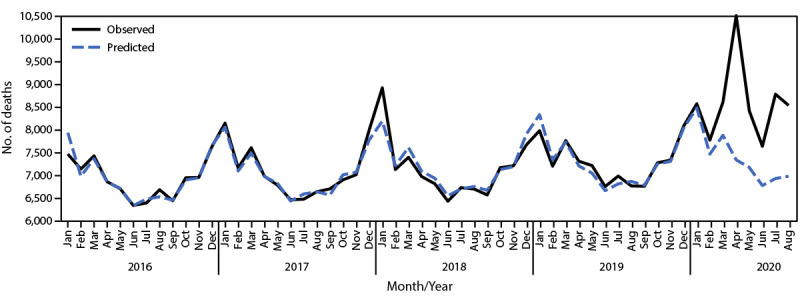
Observed* and predicted^†^ monthly deaths among patients with end-stage renal disease — United States, January 1, 2016–August 31, 2020 **Abbreviation:** CROWNWeb = Consolidated Renal Operations in a Web-Enabled Network. * Based on CROWNWeb data from January 1, 2016 through August 31, 2020. ^†^ Based on a model fit with monthly data from January 1, 2016 through December 31, 2019.

For the subgroup analyses of dialysis and transplant patients, as of February 2020, a total of 541,932 dialysis patients and 256,671 transplant patients were identified in CROWNWeb; eight patients were excluded because of missing data. Nationwide, among dialysis patients, an estimated 10.8–16.6 excess deaths per 1,000 patients (5,860–9,019 excess deaths) occurred, and among kidney transplant patients, an estimated 2.6–5.5 excess deaths per 1,000 patients (663–1,403 excess deaths) occurred.

The three ESRD Network service areas with the highest estimated number of excess deaths per 1,000 patients were Network 2 (New York), Network 3 (New Jersey, Puerto Rico, and U.S. Virgin Islands), and Network 14 (Texas) (Figure 2). This is consistent with CDC data indicating that during late January–October 2020, the largest number of COVID-19–associated deaths occurred in California, New Jersey, New York, and Texas^¶¶^ (*8*). Substantial variation among ESRD Network service areas in the temporal pattern of excess death was observed. For example, in the Network 2 service area, an increase in excess deaths was observed during March–May; however, very few or none occurred in later months, depending on the estimate (Figure 3). In contrast, in the Network 14 service area, where the initial peak of COVID-19 cases occurred later, excess deaths increased more gradually until July. In some Network service areas, such as Network 16 (Alaska, Idaho, Montana, Oregon, and Washington), few excess deaths were identified over the entire observation period. The observation of fewer excess deaths per 1,000 ESRD patients in regions affected later in the pandemic is consistent with studies of excess deaths in the overall U.S. population (*9*).

**FIGURE 2:**
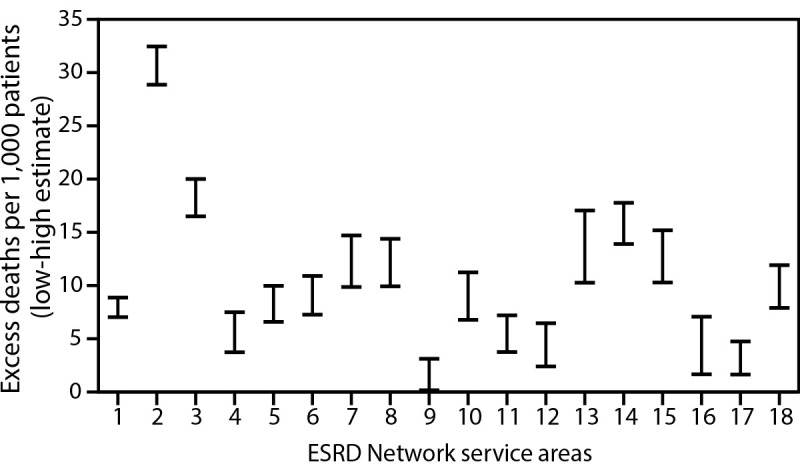
Range of high* and low^†^ estimates of excess deaths per 1,000 ESRD patients,^§^ by ESRD Network service area^¶^ — United States, February 1–August 31, 2020 **Abbreviation:** ESRD = end-stage renal disease. * High estimates were calculated as the difference between the observed number of deaths and the predicted number of deaths from the model, divided by the number of prevalent ESRD patients as of February 1, 2020. ^†^ Low estimates were calculated in a similar manner but used the upper end of the one-sided 95% prediction interval from the model in place of the mean model prediction. ^§^ Networks 2, 3, and 14 had the highest estimated number of excess deaths per 1,000 patients. ^¶^ *Network 1*: Connecticut, Maine, Massachusetts, New Hampshire, Rhode Island, and Vermont; *Network 2*: New York; *Network 3*: New Jersey, Puerto Rico, and U.S. Virgin Islands; *Network 4*: Delaware and Pennsylvania; *Network 5*: District of Columbia, Maryland, Virginia, and West Virginia; *Network 6*: Georgia, North Carolina, and South Carolina; *Network 7*: Florida; *Network 8*: Alabama, Mississippi, and Tennessee; *Network 9*: Indiana, Kentucky, and Ohio; *Network 10*: Illinois; *Network 11*: Michigan, Minnesota, North Dakota, South Dakota, and Wisconsin; *Network 12*: Iowa, Kansas, Missouri, and Nebraska; *Network 13*: Arkansas, Louisiana, and Oklahoma; *Network 14*: Texas; *Network 15*: Arizona, Colorado, Nevada, New Mexico, Utah, and Wyoming; *Network 16*: Alaska, Idaho, Montana, Oregon, and Washington; *Network 17*: American Samoa, Guam, Hawaii, Northern Mariana Islands, and Northern California; *Network 18*: Southern California.

**FIGURE 3:**
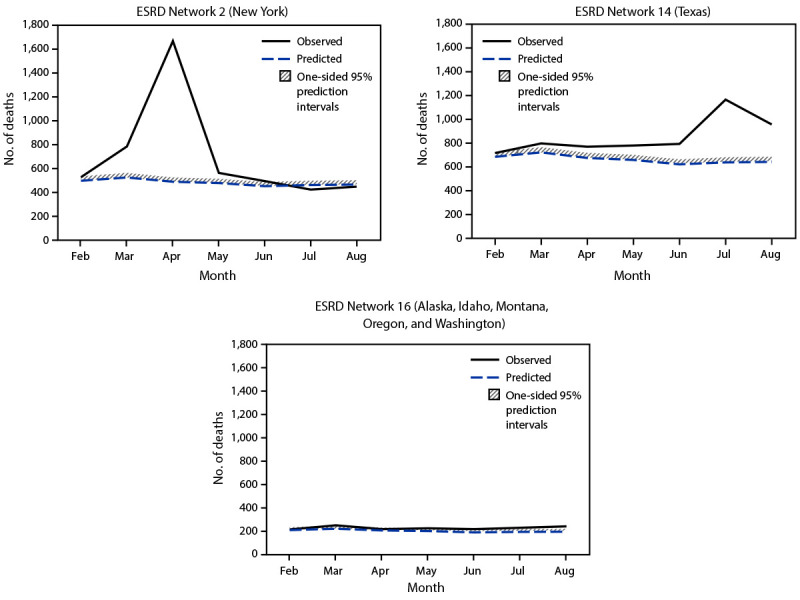
Patterns in observed* and predicted^†^ monthly deaths in the ESRD population from selected ESRD Network service areas — United States, February 1, 2020–August 31, 2020 **Abbreviations:** CROWNWeb = Consolidated Renal Operations in a Web-Enabled Network; ESRD = end-stage renal disease. * Observed number of monthly deaths was based on CROWNWeb discharge records. ^†^ Predicted number of monthly deaths was based on a model fit with data from 2016–2019. One-sided 95% prediction intervals for the model were also calculated.

## Discussion

Over a 7-month period during the early months of the COVID-19 pandemic (February–August 2020), an estimated 6,953–10,316 excess deaths occurred among ESRD patients. The estimated number of excess deaths per 1,000 patients and total excess deaths were two to three times higher among dialysis patients than among kidney transplant patients. The reasons for excess deaths in the ESRD population might include the unmet need for in-person health services or SARS-CoV-2 transmission from other patients, staff members, or the wider community during the COVID-19 pandemic. Further research into the difference in excess deaths between dialysis and kidney transplant patients is needed.

Since March 2020, all 18 ESRD Networks have implemented interventions to slow transmission of SARS-CoV-2, the virus that causes COVID-19 (*10*). Prevention messages were distributed by the ESRD Networks and ESRD NCC to facilities and patients, highlighting CDC recommendations and addressing factors that might increase patient risk, such as living in multigenerational housing.*** Using the COVID-19 dashboard created by ESRD NCC, the ESRD Networks identified facilities in regions with the most rapid growth in new cases for targeted interventions, and the ESRD Networks provided more than 4,800 instances of one-on-one technical assistance to those facilities during August–November 2020. Data-driven technical assistance has guided the implementation of processes and education initiatives to mitigate the spread of COVID-19 in dialysis facilities. Further research will be required to determine the impact of the technical assistance on excess deaths in the larger context of patient risk factors and regional variations in the progression of the pandemic.

Analyses in this report provide a rapid means for assessing the impact of the pandemic while the documentation methods, such as *International Classification of Diseases, Tenth Revision, Clinical Modification* codes (e.g., U07.1, 2019-nCoV acute respiratory disease) and clinical data workflow, for COVID-19–associated morbidity and mortality were emerging. With additional infection waves occurring in different parts of the United States during summer 2020, the reduction in COVID-19–associated excess deaths among ESRD patients during this period is worth noting. Excess deaths varied widely by ESRD Network service area and over time. The highest numbers of excess deaths per 1,000 patients were observed in regions affected early in the pandemic, with most excess deaths occurring during the first 4 months of the observation period. These patterns were generally consistent with known areas of high COVID-19 transmission in the early phase of the pandemic. Some regions had very few excess deaths, possibly because of less exposure of ESRD patients or effectiveness of early responses to the pandemic. Data on these patterns and an understanding of the mechanisms driving them could guide the planning and implementation of interventions.

The findings in this report are subject to at least four limitations. First, the CROWNWeb admission and discharge records were used as the sole data source for mortality events in this analysis. Although CROWNWeb is representative of the U.S. ESRD population, inferences from this study are limited to data included in this registry. Second, studies of excess death often correct for the lag in reporting in later periods (*7*). This correction was not possible with data available for the present study, and lags in the reporting of deaths might have resulted in undercounting of deaths in the last months of the observation period. Third, this study does not examine the effect of race and ethnicity on excess deaths, which might confound comparisons between excess death estimates in the ESRD population and the general population. Finally, the analysis presented here estimates the number of excess deaths during February 1–August 31, 2020, compared with expectations based on previous years. This observation period coincides with the early months of the COVID-19 pandemic, but the actual cause of death and the relationship to COVID-19 was not determined.

The findings of this report suggest that deaths among ESRD patients during the early phase of the pandemic exceeded those that would have been expected based on previous years’ data. Geographic and temporal patterns of excess mortality should be considered during planning and implementation of interventions, such as COVID-19 vaccination, infection control guidance, and patient education. These findings underscore the importance of data-driven technical assistance and further analyses on the causes and patterns of excess deaths in ESRD patients.

**Summary**

**What is already known about this topic?**

Patients with end-stage renal disease (ESRD) are at increased risk for COVID-19–associated morbidity and mortality.

**What is added by this report?**

Based on the national trend in ESRD deaths during the first 7 months of the U.S. COVID-19 pandemic (February 1–August 31, 2020), an estimated 8.7–12.9 excess deaths per 1,000 patients or 6,953–10,316 excess deaths in a population of 798,611 U.S. ESRD patients occurred.

**What are the implications for public health practice?**

Geographic and temporal patterns of excess mortality, including those among persons with ESRD, should be considered during planning and implementation of interventions, such as COVID-19 vaccination, patient education, and rollout of infection control guidance and technical assistance.

## Footnotes

* Renal replacement therapy is the broad name for any of the treatment choices available when kidney function has declined below an estimated glomerular filtration rate (eGFR) of 15 mL/min/1.73 m^2^. These therapies include conservative management, peritoneal dialysis, hemodialysis, and transplant. https://www.niddk.nih.gov/health-information/professionals/clinical-tools-patient-management/kidney-disease/identify-manage-patients/manage-ckd/prepare-kidney-replacement-therapy

† The ESRD Network Program (https://www.cms.gov/Medicare/End-Stage-Renal-Disease/ESRDNetworkOrganizations), which is directed by CMS, was established to improve cost-effectiveness, ensure quality of care, encourage kidney transplant and home dialysis, and support patients returning to work. To coordinate these efforts, CMS contracts with ESRD Network organizations, arranged into 18 service areas. During the COVID-19 pandemic, ESRD Networks have been charged with implementing COVID-19 mitigation strategies for dialysis facilities, kidney transplant hospitals, and ESRD patients, with technical support from ESRD NCC.

§ *Network 1*: Connecticut, Maine, Massachusetts, New Hampshire, Rhode Island, and Vermont; *Network 2*: New York; *Network 3*: New Jersey, Puerto Rico, and U.S. Virgin Islands; *Network 4*: Delaware and Pennsylvania; *Network 5*: District of Columbia, Maryland, Virginia, and West Virginia; *Network 6*: Georgia, North Carolina, and South Carolina; *Network 7*: Florida; *Network 8*: Alabama, Mississippi, and Tennessee; *Network 9*: Indiana, Kentucky, and Ohio; *Network 10*: Illinois; *Network 11*: Michigan, Minnesota, North Dakota, South Dakota, and Wisconsin; *Network 12*: Iowa, Kansas, Missouri, and Nebraska; *Network 13*: Arkansas, Louisiana, and Oklahoma; *Network 14*: Texas; *Network 15*: Arizona, Colorado, Nevada, New Mexico, Utah, and Wyoming; *Network 16*: Alaska, Idaho, Montana, Oregon, and Washington; *Network 17*: American Samoa, Guam, Hawaii, Northern Mariana Islands, and Northern California; *Network 18*: Southern California.

¶ On November 9, 2020, CMS launched the ESRD Quality Reporting System, which merged three legacy systems, including CROWNWeb, into one ESRD program.

** Bootstrapping was used to generate the 95% prediction interval for the predicted mean number of deaths. One hundred replicate samples of the original data were obtained by sampling with replacement. Model coefficients were then estimated, and the predicted mean number of deaths was obtained. For each bootstrap replicate, 100 predictions were randomly generated from a Poisson distribution, with the mean equal to the predicted mean number of deaths for that bootstrap replicate. The 95th percentile of the resulting 10,000 simulated observations was then taken as the upper end of the one-sided 95% prediction interval.

†† 45 C.F.R. part 46.102(l)(2), 21 C.F.R. part 56; 42 U.S.C. Sect. 241(d); 5 U.S.C. Sect. 552a; 44 U.S.C. Sect. 3501 et seq.

§§ In this study, patients receiving dialysis and patients with a previous failed kidney transplant who subsequently required chronic dialysis treatment are categorized as “dialysis patients.” Patients with a successful kidney transplant, even if temporary dialysis is required after transplantation, are categorized as “kidney transplant patients.”

¶¶ California is divided into two ESRD Network service areas (17 and 18); therefore, the impact on that entire state is not apparent in this analysis.

*** The ESRD Networks and ESRD NCC distributed information highlighting CDC recommendations regarding mask use, physical distancing, and handwashing. Resources were also distributed to address factors that might increase patient risk. ESRD NCC developed materials including reminders for dialysis patients, a guide to using telemedicine, and tips for mental health care.
